# Chemical composition, characteristics concerned with fermentative quality and microbial population of ensiled pearl millet and sorghum stover in semi‐arid West Africa

**DOI:** 10.1111/asj.13463

**Published:** 2020-11-22

**Authors:** Yimin Cai, Zhumei Du, Seishi Yamasaki, Delma B. Jethro, Nignan Man

**Affiliations:** ^1^ Japan International Research Center for Agricultural Sciences (JIRCAS) Tsukuba Japan; ^2^ College of Grassland Science and Technology China Agricultural University Beijing PR China; ^3^ Institute of Environment and Agriculture Research (INERA) Koudougou Burkina Faso

**Keywords:** chemical composition, crop stover, field exposure, semi‐arid Africa, silage

## Abstract

To effectively utilize crop by‐product resources for ruminant feed in semi‐arid West Africa, we studied the chemical composition, characteristics concerned with fermentative quality, and microbial population of ensiled pearl millet stover (PMS) and sorghum stover (SS) in Mozambique. After panicle harvest, the PMS and SS were exposed in the field for 7, 21, 35, 49, 63, 77, 91, and 120 days under natural weather conditions. The fresh stover silages were prepared and stored for 120 days. With increased exposure time, the dry matter, neutral detergent fiber, acid detergent lignin, and neutral detergent insoluble nitrogen contents increased, whereas the crude protein, ether extract, gross energy, digestible energy, metabolizable energy, and true protein contents decreased. After 120 days of field exposure, aerobic bacteria dominated both stovers, and lactic acid bacteria (LAB) decreased to below detectable levels. After 120 days of ensiling, LAB dominated the silage of both crops, while the harmful microorganisms as aerobic bacteria, coliform bacteria, yeast, and mold were reduced or below detectable levels. Both silages did not produce more lactic acid to reduce the pH value, but they preserved nutrients well during ensiling. Therefore, PMS and SS can be prepared as silage for ruminant feed in semi‐arid West Africa.

## 1 INTRODUCTION

A mixed farming system for smallholder farm in Africa generally consists of integrated crop and livestock production activities. In this system, grains are important staple foods for the local people, and the crop by‐products are the main sources of roughage for ruminants, including cows, sheep, and goats in West Africa (Cai et al., 2020; Fernández‐Rivera et al., 2005). The most important limiting factor for cows in the tropics is feed limitation in terms of quantity and quality, especially during the dry season (Cai et al., 2020; Khota et al., 2017). Cows that cannot access high‐quality roughage produce reduced amounts of milk and meat (Wiedmeier et al., 2002). Some varieties of crops that exhibit good tolerance to hot weather and drought conditions have been developed recently, and their adaptability to various cultivation conditions, the nutritive value of the crop by‐products, and their contribution to animal productivity have been studied in West Africa (Martin et al., 2017).

Pearl millet (*Pennisetum glaucum* L.) and sorghum (*Sorghum bicolor* [L.] Moench) sustain millions of rural people in the semi‐arid tropics of Africa and Asia. Thus, improvements in the production, storage, utilization, and consumption of these food crops will greatly contribute to the food security and nutrition of the residents of these areas (FAO, 1995). Pearl millet and sorghum are indigenous African cereals that, unlike maize and wheat, are well‐adapted to African semi‐arid and sub‐tropical agronomic conditions. Overall, 49 and 55% of the world's pearl millet and sorghum cultivation areas, respectively, are in Africa. These grains represent the major source of dietary energy and protein for approximately 1 billion people in the semi‐arid tropics. They are well‐adapted to environments with limited rainfall and high temperatures, and can even thrive in soils with low nutrient and moisture contents (Singh et al., 2017). In fact, their by‐products are considered among the globally important crop by‐products produced in Africa. However, the by‐products are generally discarded in the fields, where they are often burned and used as fertilizer (Hauser et al., 2006).

Both pearl millet stover (PMS) and sorghum stover (SS) could be used as feed for livestock because they are cheap and abundant in the tropics, where other green fodder is unavailable. The PMS and SS can also be preserved as hay. However, there are some disadvantages to using hay made from these crops. For example, local farmers usually begin storing crop stover or native grass hay in the early dry season, dry matter (DM) and crude protein (CP) are generally lost during the drying and storage process (Scarbrough et al., 2002). Both stover types can be prepared as silage. This form of preservation allows for the qualitative characteristics of the forage to be preserved as well, and guarantee that herds will have feed stocks when pasture supplies are low (Amer et al., 2012).

However, how does the preserving forms influence the chemical composition of stover exposed in the field, and the fermentation dynamics as silage stored in silos. In order to effectively utilize the crop by‐product resources for ruminant feed, we studied the chemical composition of PMS and SS during field exposure, and the characteristics concerned with fermentative quality and microbial population of silages in semi‐arid West Africa.

## 2 MATERIALS AND METHODS

### 2.1 Materials and experimental design

**Figure 1:**
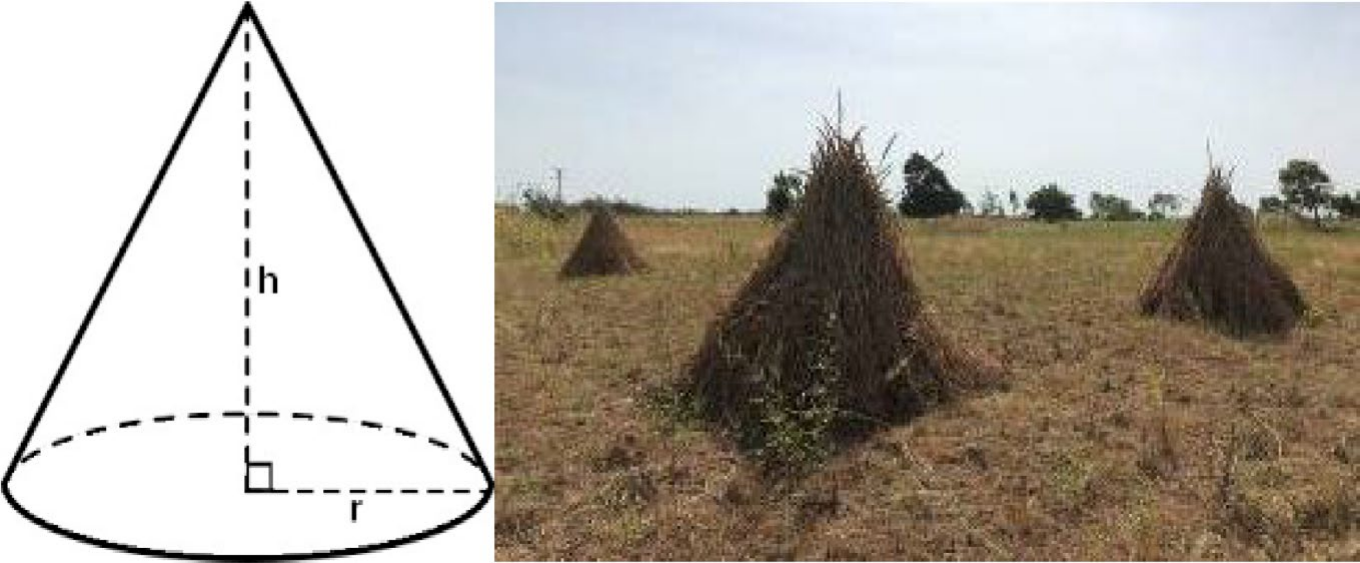
Schematic diagram of stover stacking method (Left). PMS for sampling were stacked into three conical mountains of similar size (each approximately 700 kg, 1 m radius × 2 m height) in the field of a local farm without any plastic sheet covering (Right). SS were stacked in the same way. PMS, pearl millet stover; SS, sorghum stover

The pearl millet and sorghum that are widely cultivated in West Africa were selected for use in this experiment. Both stovers including stems and leaves were harvested at maturity stage in a local farm field, Koudougou, Burkina Faso on October 27, 2018. The collected stover materials were divided into three fractions. The first fraction was sampled to characterize the two kinds of stovers used as fresh crop. The second fraction was designed for dry stover making and sampling. As shown in Figure 1, stover bundles of each crop were piled up in three conical stover mountains, on the field without a cover of plastic sheeting following the drying storage method of the local farms. The mountains of stovers were exposed to the natural weather environment in the field for drying, and a mixture of outer and center layers of each stover mountain were taken as samples after exposure times of 7, 21, 35, 49, 63, 77, 91, and 120 days. During the experiment, the average temperature was 31.3°C, and the average air humidity was 15.8%. The third fraction was used for making silage, for which a laboratory‐scale fermentation system was employed (Cai et al., 2020).

After harvest, fresh PMS and SS (each type, 40 kg) were immediately cut into approximately 1–2 cm lengths before ensiling using a chopper (130DX; ARS Co., Ltd, Osaka, Japan). About 40 kg of each homogenous crop type was randomly divided into five equal parts, and then approximately 8 kg of material was packed separately into one 20 L polyethylene drum (Hiryu KN type; Asahikasei, Tokyo, Japan) silo for each replicate. Three silos were prepared for each treatment. All silos were kept at ambient temperature (25–38°C). After 120 days of fermentation, three of five silos were randomly opened to analyze the chemical composition, fermentation quality, microbial population, macro mineral, and protein composition.

To compare the differences in chemical composition during exposure and ensiling, fresh, dry, and ensiled stover samples were collected. The fresh PMS and SS stovers samples were collected on the day of cutting. The dry stover samples were collected after 120 days of drying. The ensiled stover samples were collected after 120 days of ensiling. The chemical composition of the fresh, dry, and ensiled samples was determined in triplicate after 120 days of fermentation.

### 2.2 Chemical composition and energy analysis

The pre‐ensiled, field‐exposed, and ensiled PMS and SS samples were oven‐dried at 70°C for 48 hr. After drying, the samples were ground to through a high speed vibrating sample mill model (T1‐200, for use with two containers of working capacity 50 ml; CMT Co., Ltd, Fukushima, Japan) for chemical composition analysis. Samples were analyzed according to the method of the Association of Official Analytical Chemist (AOAC, 2000) for DM (method 930.15), ash (method 923.03), CP (method 990.03), and ether extract (EE, method 920.39), respectively. The residual moisture was removed by drying additionally at 105°C for at least 12 hr, and chemical composition were measured relative to DM content. Determination of sample ash from 1.0 g dried powder samples was done by burning in a muffle furnace at 550°C overnight in a muffle furnace (Thermolyne F30420C; Thermo Scientific, Asheville, NC, USA). The organic matter (OM) content was calculated as the weight lost after ashing. Nitrogen (N) content was measured by Kjeldahl method using a Kjeldahl nitrogen/protein analyzer (Model 1,026; Foss Technol. Co., Ltd, Hillerod, Sweden). N was converted to CP by using a conversion factor of 6.25. Neutral detergent fiber (NDF), acid detergent fiber (ADF), and acid detergent lignin (ADL) were determined as described by Van Soest et al. (1991). Both NDF and ADF were expressed exclusive of residual ash; heat stable amylase and sodium sulphite were used for the NDF procedure. ADL was analyzed by solubilization of cellulose with sulfuric acid. Lactic acid buffer capacity **(**LBC) was determined by titrating with NaOH from pH 4.0 to 6.0 (mmol/kg DM) after first reducing pH below 4.0 using HCl as described by Muck et al. (1991). Gross energy (GE) was determined using an automatic oxygen bomb calorimeter (CA‐4PJ; Shimadzu, Kyoto, Japan). Digestible energy (DE) and metabolizable energy (ME) were calculated using the following formulas on the basis of the National Research Council (Gao et al., 2019; NRC, 1996):

DE=GE×[70.19‐1.364×(ADF‐29.83)‐3.94+0.104×CP+0.149×EE+0.022×NDF‐0.244×ash]/100

ME=0.82×DE

Herein, CP, EE, NDF, ADF, and ash are expressed as a percentage of DM (% of DM), and the unit for GE, DE, and ME is MJ/kg of DM.

### 2.3 Protein and macro mineral composition analysis

True protein (TP) and neutral detergent insoluble nitrogen (NDIN) were determined using methods described by Cai et al. (2020). All samples concentrations for macro mineral analysis of calcium (Ca), phosphorous (P), magnesium (Mg), and potassium (K) were prepared using a wet‐ashing method as described by Hollis et al. (2019), and then analyzed using an atomic absorption spectrophotometer (LAMBDA 1,050; PerkinElmer, Shelton, CT, USA).

### 2.4 Analysis of silage fermentation

The fermentation products of the PMS and SS silages were analyzed using cold‐water extracts. The remaining wet silage sample (10 g) was homogenized in 90 ml of sterilized distilled water and kept in a refrigerator at 4°C for 24 hr as described by Cai (2004). Thereafter, the extract samples were filtered through quantitative ashless filter paper (circle size: 5A, 110 mm; Advantec Co., Ltd, Tokyo, Japan). The filtrate was used to determine pH, ammonia *N* (NH_3_‐N), and organic acid (lactic acid, acetic acid, propionic acid, and butyric acid) contents. The pH was measured using a glass electrode pH meter (D‐71; Horiba Co., Ltd, Kyoto, Japan). The NH_3_‐N content of silages were determined by the steam distillation of the filtrates as described by Cai (2004) using the Kjeltech auto distillation (2,200; Foss Tecator, Hoganas, Sweden). The silage filtrates were shaken with cation exchange resin (Amberlite, IR 120B H AG; Organo Corporation, Tokyo, Japan) and centrifuged at 6,500 × *g,* 4°C for 5 min. The supernatants were passed through a 0.45‐mm filter under pressure, and the filtrates were then injected into an high‐performance liquid chromatography (HPLC, LC‐2000 plus; JASCO Corpration, Tokyo, Japan) to determine organic acid contents in accordance with the methods described by Cai (2004). The analysis conditions were as follows: column, Shodex RS pak KC‐811 (8.0 mm × 30 cm; Showa Denko K. K. Tokyo, Japan); oven temperature, 60°C; detector, Jasco UV‐2070, 450 nm; eluent, 3 mmol/L HClO_4_ 1.0 ml/min; reagent, 0.2 mmol/L bromothymol blue + 8 mmol/L Na_2_HPO_4_ + 2 mmol/L NaOH, 1.0 ml/min.

### 2.5 Microbial analysis

The microbial population of lactic acid bacteria (LAB), aerobic bacteria, coliform bacteria, yeasts, and molds of fresh forages and silages were analyzed using plate count method as described by Cai et al. (1999). For each sample, 10 g of material was added to 90 ml of sterilized saline solution (8.50 g/L NaCl) and homogenized for 5 min in a Stomacher laboratory blender (SH‐IIM; Elmex Co., Ltd, Tokyo, Japan). The resulting suspension was serially diluted from 10^−1^ to 10^−5^ in saline solution. A 0.05 ml aliquot from each diluted suspension was spread onto agar plates. LAB were counted on Lactobacilli de Man, Rogosa, and Sharpe (MRS) agar medium (Difco Laboratories, Detroit, MI, USA) in an anaerobic box (ANX‐1; Hirosawa Co., Ltd, Tokyo, Japan). Aerobic bacteria were grown on nutrient agar medium (Nissui‐Seiyaku Co., Ltd, Tokyo, Japan) under aerobic conditions. Coliform bacteria were counted on blue light agar medium (Nissui‐Seiyaku Co., Ltd, Tokyo, Japan). Yeasts and molds were counted on potato dextrose agar medium (Nissui‐Seiyaku Co., Ltd, Tokyo, Japan) with sterilized tartaric acid solution (pH 3.5). Yeasts were distinguished from molds and other bacteria based on colony appearance and cell morphology. All ager plates were incubated at 30°C for 2–3 days, and the microbial counts were counted as viable numbers of microorganisms and expressed in colony‐forming units (cfu) per gram of fresh matter (FM).

### 2.6 Statistical analyses

Analysis of variance (ANOVA) was performed using the general linear model procedure of SAS (rev. 9.4; Institute Inc., Cary, NC, USA). The chemical composition, energy, protein, macro mineral, fermentation quality, and microbial population data were subjected to one‐way ANOVA. The statistical difference between the means was determined using Tukey's multiple comparisons, the level of considered significance was set to 5%.

## 3 RESULTS

### 3.1 Chemical composition and energy of stovers during field exposure

Changes in the chemical composition and energy in PMS and SS during field exposure are shown in Table 1. At 7 days of field exposure, the DM values of the PMS and SS were 56.87 and 73.01%, respectively. After 21 days of field exposure, the DM values of both stovers reached >93.56%. The CP, EE, GE, DE, and ME values decreased (*p* < .001) with exposure time, whereas NDF, ADF, and ADL values increased (*p* < .05) in both stover types, the OM content in both types of stovers did not change markedly. At 91 days of exposure, the CP and NDF of the PMS and SS changed to 2.58 and 1.65% and to 82.20 and 82.17%, respectively.

**Table 1:** Chemical composition and energy in PMS and SS during field exposure^1^

Items	Exposure day	DM	Chemical composition (% of DM)						Energy (MJ/kg of DM)		
		(%)	OM	CP	EE	NDF	ADF	ADL	GE	DE	ME
PMS	7	56.87^d^	93.10	3.89^a^	0.77^a^	78.27^b^	49.10^e^	8.09^e^	18.85^a^	9.00^a^	7.38^a^
	21	93.56^c^	93.09	3.05^b^	0.60^b^	79.92^ab^	50.99^d^	8.92^d^	18.80^a^	8.07^b^	6.62^b^
	35	97.32^b^	92.99	2.81^c^	0.46^c^	80.50^ab^	51.74^c^	9.69^c^	18.73^a^	7.68^c^	6.29^c^
	49	97.54^b^	92.78	2.68^d^	0.49^d^	81.51^ab^	53.62^b^	10.51^b^	18.71^a^	7.57^cd^	6.21^c^
	63	99.05^a^	92.70	2.62^de^	0.28^e^	81.89^a^	54.52^a^	10.26^b^	18.38^b^	7.30^e^	5.99^d^
	77	98.80^a^	92.59	2.68^d^	0.29^e^	82.00^a^	54.27^a^	11.58^a^	18.13^b^	7.43^de^	5.79^e^
	91	97.76^b^	92.50	2.58^e^	0.28^e^	82.20^a^	54.19^a^	11.69^a^	17.99^c^	7.36^e^	5.50^f^
	*SEM*	0.14	0.50	0.03	0.01	0.05	0.15	0.11	0.06	0.06	0.06
	*p*‐value	<.001	.959	<.001	<.001	.038	<.001	<.001	<.001	<.001	<.001
SS	7	73.01^e^	94.57	2.14^a^	0.91^a^	79.27^c^	50.83^b^	9.08^f^	18.91^a^	8.05^a^	6.60^a^
	21	94.32^d^	94.59	2.08^a^	0.85^b^	80.10^bc^	51.64^ab^	9.16^f^	18.80^a^	7.01^b^	6.18^b^
	35	96.74^c^	94.44	1.92^b^	0.84^b^	81.16^abc^	52.99^a^	9.34^e^	18.36^bc^	6.57^c^	5.79^c^
	49	97.03^c^	94.45	1.87^b^	0.80^c^	81.95^ab^	53.02^a^	10.49^d^	18.35^bc^	6.45^c^	5.70^cd^
	63	99.51^a^	94.29	1.86^b^	0.72^d^	82.06^a^	53.07^a^	10.82^c^	18.42^b^	6.49^c^	5.54^de^
	77	98.01^b^	94.28	1.63^c^	0.73^d^	82.27^a^	53.37^a^	11.39^b^	18.23^c^	6.50^c^	5.48^ef^
	91	99.44^a^	94.19	1.65^c^	0.74^d^	82.17^a^	53.26^a^	11.93^a^	18.01^d^	6.47^c^	5.31^f^
	*SEM*	0.11	0.27	0.02	0.01	0.60	0.54	0.03	0.04	0.06	0.06
	*p*‐value	<.001	.921	<.001	<.001	.018	.033	<.001	<.001	<.001	<.001

Note

The lactic acid buffer capacity (LBC) content of fresh PMS and SS were 2,292.53 and 3,013.93 meq/kg of DM, respectively.

^a–g^Different lowercase letters within a column among exposure days indicate significant differences at p < .05. Each value is the mean of three samples. SEM, standard error of the mean.

^1^ DM, dry matter; OM, organic matter; CP, crude protein; EE, ether extract; NDF, neutral detergent fiber; ADF, acid detergent fiber; ADL, acid detergent lignin; GE, gross energy; DE, digestible energy; ME, metabolizable energy; PMS, pearl millet stover; SS, Sorghum stover.

### 3.2 Protein and macro mineral composition of stovers during field exposure

The protein and macro mineral composition in PMS and SS during field exposure are shown in Table 2. During 7–49 days of field exposure, the TP content decreased (*p* < .001) continuously, whereas the NDIN levels increased (*p* < .001) in both stovers. After 63 days of field exposure, the protein composition tended to be stable, without large changes. Regarding macro mineral composition, Ca, P, Mg, and K contents in both types of stovers did not change markedly during field exposure duration.

**Table 2:** Protein and macro mineral composition in PMS and SS during field exposure^1^

Items	Exposure day	Protein (% of CP)			Macro mineral (g/kg of DM)			
		NDIN	TP		Ca	P	Mg	K
PMS	7	38.77^e^	60.86^a^		0.23	0.22	0.25	2.28
	21	39.23^d^	60.38^a^		0.26	0.27	0.17	2.21
	35	39.58^c^	59.86^a^		0.28	0.32	0.23	2.38
	49	40.55^b^	56.86^b^		0.21	0.23	0.12	2.50
	63	42.10^a^	54.85^b^		0.25	0.15	0.21	2.34
	77	42.73^a^	54.86^b^		0.25	0.10	0.34	2.33
	91	43.37^a^	54.80^b^		0.25	0.16	0.25	2.04
	*SEM*	0.08	0.76		0.01	0.01	0.01	0.03
	*p*‐value	<.001	<.001		.096	.266	.101	.081
SS	7	47.27^d^	48.95^a^		0.24	0.03	0.16	1.50
	21	47.71^c^	46.36^b^		0.27	0.04	0.29	1.24
	35	47.01^e^	45.64^bc^		0.25	0.03	0.22	1.30
	49	48.75^b^	45.51^c^		0.26	0.02	0.33	1.38
	63	51.37^a^	44.71^d^		0.30	0.03	0.31	1.37
	77	51.38^a^	44.62^d^		0.30	0.04	0.13	1.12
	91	51.41^a^	44.50^d^		0.21	0.03	0.15	1.14
	*SEM*	0.05	0.25		0.01	0.01	0.01	0.02
	*p*‐value	<.001	<.001		.101	.124	.061	.101

^a–e^Different lowercase letters within a column among exposure days indicate significant differences at p < .05. Each value is the mean of three samples. SEM, standard error of the mean.

^1^ DM, dry matter; CP, crude protein; NDIN, neutral detergent insoluble nitrogen; TP, true protein; Ca, calcium; P, phosphorous; Mg, magnesium; K, potassium; PMS, pearl millet stover; SS, sorghum stover.

### 3.3 Comparative analysis of the chemical composition in fresh, dry, and ensiled stovers

Comparative analysis of the chemical composition of the fresh, dry stover after 120 days of exposure, and the silage after 120 days of ensiling is shown in Table 3. Before ensiling, fresh PMS and SS comprised 43.15 and 44.44% DM, respectively, their values increased to more than 98.47% in two types of dry stovers after 120 days exposure. Compared to the both fresh materials, the CP and EE contents decreased for both dry stovers, by 40%–42% and 27%–64%, and the ADL and ADF contents increased by 23%–74% and 12%–29%, respectively. For both silages, the CP and EE decreased by 5%–9% and 7%–8%, respectively, but the ADL and ADF contents did not change greatly.

**Table 3:** Chemical composition in fresh, dry, and ensiled PMS and SS^1^

Items	Preservation method	DM (%)	Chemical composition (% of DM)					
			OM	CP	EE	NDF	ADF	ADL
PMS	Fresh	43.15^b^	93.37	4.18^a^	0.75^a^	77.10^b^	48.30^b^	6.89^b^
	Dry	99.17^a^	92.49	2.46^b^	0.27^b^	82.10^a^	54.25^a^	11.94^a^
	Ensiled	43.77^b^	93.17	3.93^a^	0.69^a^	77.74^b^	48.67^b^	6.91^b^
	*SEM*	0.56	0.53	0.09	0.03	0.42	0.37	0.10
	*p*‐value	<.001	.516	<.001	<.001	<.001	<.001	<.001
SS	Fresh	44.44^b^	94.22	2.49^a^	0.99^a,b^	78.14^b^	49.31^b^	9.27^b^
	Dry	98.47^a^	94.10	1.48^c^	0.72^b^	82.09^a^	63.57^a^	11.43^a^
	Ensiled	44.06^b^	94.15	2.28^b^	0.92^a^	78.85^b^	49.71^b^	9.35^b^
	*SEM*	0.30	0.18	0.02	0.03	0.28	0.38	0.03
	*p*‐value	<.001	.897	<.001	.003	<.001	<.001	<.001

Note

The duration of silage storage and field exposure for which PMS and SS were both 120 days.

^1^ DM, dry matter; OM, organic matter; CP, crude protein; EE, ether extract; NDF, neutral detergent fiber; ADF, acid detergent fiber; ADL, acid detergent lignin; PMS, pearl millet stover; SS, Sorghum stover.

^a,b^ Different lowercase letters within a column among preservation methods indicate significant differences at *p* < .05. Each value is the mean of three samples. *SEM*, standard error of the mean.

### 3.4 Energy, protein, and macro mineral in fresh, dry, and ensiled stovers

The energy, protein, and macro mineral of fresh, dry, and ensiled PMS and SS are shown in Table 4. The energy, protein, and macro mineral composition did not differ markedly between fresh and ensiled materials for both PMS and SS. Compared to the fresh stovers, the TP content in the dry PMS decreased by 8.38% CP, and the NDIN contents increased by 7.49% CP. The TP content in the dry SS decreased by 9.68% CP, and the NDIN contents increased by both approximately 15% CP. The contents of DE, ME, Ca, and Mg were lower (*p* < .05); the content of P were higher (*p* < .05), the GE and K content were similar in the dry stovers of both crop types compared with fresh and ensiled stovers.

**Table 4:** Energy, protein, and macro mineral composition in fresh, dry, and ensiled PMS and SS^1^

Items	Preservation method	Energy (MJ/kg of DM)			Protein (% of CP)		Macro mineral (g/kg of DM)			
		GE	DE	ME	NDIN	TP	Ca	P	Mg	K
PMS	Fresh	19.07	10.66^a^	8.74^a^	37.24^b^	62.74^a^	0.37^a^	0.18^b^	0.29^a^	1.89
	Dry	17.66	7.38^b^	5.51^b^	44.73^a^	54.36^b^	0.24^b^	0.26^a^	0.24^b^	2.10
	Ensiled	18.96	10.56^a^	8.66^a^	37.68^b^	62.12^a^	0.39^a^	0.18^b^	0.31^a^	1.90
	*SEM*	0.85	0.27	0.26	0.48	0.17	0.01	0.01	0.01	0.02
	*p*‐value	.471	<.001	<.001	<.001	<.001	<.001	<.001	.008	.091
SS	Fresh	18.99	9.76^a^	8.00^a^	46.36^b^	52.15^a^	0.37^a^	0.03^ab^	0.29^a^	0.89
	Dry	17.86	6.41^b^	4.91^b^	61.41^a^	42.47^b^	0.22^b^	0.03^a^	0.16^b^	1.33
	Ensiled	19.04	9.70^a^	7.95^a^	47.23^b^	52.13^a^	0.36^a^	0.02^b^	0.30^a^	1.15
	*SEM*	0.70	0.23	0.17	0.26	0.09	0.03	0.01	0.01	0.01
	*p*‐value	.456	<.001	<.001	<.001	<.001	.016	.098	.021	.061

Note

The duration of silage storage and field exposure for which PMS and SS were both 120 days.

^1^ DM, dry matter; GE, gross energy; DE, digestible energy; ME, metabolizable energy; CP, crude protein; NDIN, neutral detergent insoluble nitrogen; TP, true protein; Ca, calcium; P, phosphorous; Mg, magnesium; K, potassium; PMS, pearl millet stover; SS, sorghum stover.

^a,b^ Different lowercase letters within a column among preservation methods indicate significant differences at *p* < .05. Each value is the mean of three samples. *SEM*, standard error of the mean.

### 3.5 Microbial population in fresh, dry, and ensiled stovers

The microbial population in fresh, dry, and ensiled PMS and SS are shown in Table 5. Aerobic bacteria dominated the stovers of both crops before ensiling, with counts of 10^5^–10^6^ cfu/g of FM. After 120 days of fermentation, LAB dominated the silage of both crops, with counts ranging from 10^5^ to 10^6^ cfu/g of FM. Counts of aerobic and coliform bacteria were similar and ranged from 10^3^ to 10^4^ cfu/g of FM. Counts of yeast and mold were below detectable levels (< 10 cfu/g of FM) in all stover silages. After 120 days of field exposure, LAB counts decreased to below detectable levels, and aerobic bacteria dominated both stovers with a counts of 10^4^ cfu/g of FM. Counts of coliform bacteria, yeast (10^3^ cfu/g of FM), and mold (10^2^ cfu/g of FM) were similar between stover types. LAB counts were lower (*p* < .05) in dry stover than in fresh and ensiled stovers.

**Table 5:** Microbial population of fresh, dry, and ensiled PMS and SS^1^

Items	Preservation method	Microbial population (cfu/g of FM)				
		LAB	Aerobic bacteria	Coliform bacteria	Yeast	Mold
PMS	Fresh	2.22 × 10^4b^	5.20 × 10^5^	3.53 × 10^5^	3.67 × 10^5^	9.67 × 10^3^
	Dry	ND	1.04 × 10^4^	7.05 × 10^3^	7.52 × 10^3^	1.93 × 10^2^
	Ensiled	1.67 × 10^5a^	1.75 × 10^3^	1.39 × 10^3^	ND	ND
	*SEM*	2.67	2.14	1.86	1.13	3.12
	*p*‐value	.009	.226	.376	.102	.123
SS	Fresh	3.45 × 10^4b^	2.35 × 10^6a^	3.83 × 10^5a^	4.68 × 10^5^	1.32 × 10^4^
	Dry	ND	5.29 × 10^4b^	6.97 × 10^3b^	8.57 × 10^3^	3.26 × 10^2^
	Ensiled	2.50 × 10^6a^	2.50 × 10^4b^	1.00 × 10^4b^	ND	ND
	*SEM*	1.67	0.58	2.37	1.97	0.58
	*p*‐value	<.001	.049	<.001	.249	.260

Note

The duration of silage storage and field exposure for which PMS and SS were both 120 days.

^a,b^Different lowercase letters within a column among preservation methods indicate significant differences at p < .05. Each value is the mean of three samples. SEM, standard error of the mean.

^1^ cfu, colony‐forming unit; FM, fresh matter; LAB, lactic acid bacteria; ND, not detected; PMS, pearl millet stover; SS, sorghum stover.

### 3.6 Fermentation quality of stover silages

Details of the fermentation quality of the PMS and SS silage after 120 days of ensiling are shown in Table 6. The moisture content in the PMS and SS was similar, ranging from 54.6% to 56.2%. For both stover silages, the pH value and NH_3_‐N content were < 4.65% of FM and < 0.52 g/kg of FM, respectively, whereas the lactic acid content was > 0.42% of FM. The butyric acid and propionic acid contents were below the detection level.

**Table 6:** Fermentation quality of PMS and SS silages after 120 days of ensiling^a^

Items^1^	Treatments	
	PMS	SS
Moisture (%)	56.23	54.59
pH	4.65	4.28
Lactic acid (% of FM)	0.42	0.57
Acetic acid (% of FM)	0.44	0.43
Propionic acid (% of FM)	ND	ND
Burytic acid (% of FM)	ND	ND
NH_3_‐*N* (g/kg of FM)	0.52^a^	0.45^b^

Note

Each value is the mean of three samples.

^a^ FM, fresh matter; ND, not detected; NH_3_‐*N*, ammonia nitrogen; PMS, pearl millet stover; SS, sorghum stover.

## 4 DISCUSSION

Pearl millet and sorghum have been important staples in West Africa for centuries, and their dry stovers are still the principal sources of feed for dairy cows in these regions. Generally, after grain harvest, as field exposure time increases, the stover loses more moisture, but the fiber and lignin contents increase substantially (McEniry et al., 2014). These results were also observed in this study. Higher levels of NDF, ADF, and ADL were observed in the PMS and SS, resulting in decreased DE, which in turn was negatively correlated with cell wall structural components such as cellulose and lignin. Gao et al. (2019) reported that rain, sunlight, and microbial growth during field exposure may contribute to a decline in CP and energy contents as exposure time is prolonged. In this study, CP and TP contents decreased significantly, and NDIN increased significantly in the PMS and SS with increased exposure time (Tables 1, 2) due to the loss of feed nutrients such as non‐cellulosic saccharides. Aerobic bacteria present in stover, which cause spoilage, and plant respiration consume nutrients such as proteins and sugars in plants (Pordesimo et al., 2005). Aerobic bacteria are the dominant microbial population in the two stovers during exposure in the field. These processes likely contributed significantly to the decreased DE observed in this study. Minerals are the main elements that constitute animal tissues and maintain the normal physiological functions and biochemical metabolism of livestock (Ammerman & Goodrich, 1983). Pearl millet and sorghum stovers are important roughage sources for ruminants in Africa, but a limited information is available on mineral composition of pearl millet and sorghum. In this study, some mineral content of two stovers were different, and they also differed among fresh materials, silage, and dry stover, which indicating that the analysis data need to be fully considered in the design of ruminant feed formulations, and these information will be useful for animal production in Africa.

In this study, the moisture contents of the fresh PMS and SS were lower than 56.85%, and the chemical composition did not change greatly between 63 and 120 days of exposure. Both fresh stovers with low moisture could be used to prepare silage, which can inhibit clostridia fermentation. However, once the moisture becomes less than 40%, the fermentation of LAB in the silage fermentation will be restricted (Cai et al., 2020). In this case, it is necessary to pay close attention to take measures to adjust the stover moisture for silage making.

In the dry season, livestock grazing in the field feed entirely on dry stovers with high ADL and ADF contents, resulting in very low DM intake and low productivity. Poor nutrition for livestock results in low rates of reproduction and production, as well as increased susceptibility to disease. Large quantities of crop residues, including PMS and SS, are left in the field each year, where they are wasted. Thus, it is important to find ways to reverse this situation by adapting familiar, simple, and effective technologies to local conditions and by introducing new approaches for improving the use of crop residues and low‐quality fibrous feeds. Relying on effective agricultural techniques, efforts should also be made to increase the feed and livestock production base. Silage production using the local available crop by‐products can be considered a good strategy for addressing the feed shortage for ruminants in West Africa. Both stovers in the present study can be prepared as high‐quality silages, which can be preserved well for the long ensiling period of 120 days. Therefore, silage prepared with crop stovers, as a fermented storage feed, can be used effectively in local livestock production, thereby improving the nutrition of livestock and making up for the feed shortage.

To comprehensively elucidate the microbial composition in fresh, dry, and ensiled PMS and SS, we analyzed the abundances of five types of microbes related to silage fermentation‐LAB, aerobic bacteria, coliform bacteria, molds, and yeasts. When LAB are present in low numbers in crop forage, or when grasses fail to produce sufficient lactic acid during fermentation, the growth of harmful bacteria is not inhibited sufficiently, resulting in poor‐quality silage. With an abundance of at least 10^5^ cfu/g of FM of epiphytic LAB, good‐quality silage can be produced (Bureenok et al., 2005; Cai et al., 1999). In this study, low LAB counts and high counts of aerobic bacteria, coliform bacteria, and yeasts were observed in both types of fresh stovers before ensiling, implying that silage fermentation should include the use of microbial inoculant. However, the LAB counts increased continuously through 120 days of fermentation, and these bacteria remained dominant over other microorganisms. Coliform bacteria and molds in all silages were below detectable levels. As shown in Table 5, we also found that the microbial composition differed between the two crop types. The cultivation environment and chemical composition of both crops, especially moisture and chemical composition, may influence the distribution of native microorganisms (Wang et al., 2019). All dry stovers had much lower microbe counts than did fresh stovers or silages. This may because water activity is an important factor affecting the survival of microorganisms on the plant surfaces (Valero et al., 2016). The water activity decreased during field exposure and could not meet the basic living environment requirement for the growth and development of bacteria. Therefore, with increased time of continuous exposure and consequent decrease in moisture, large reductions in the counts of all microorganisms associated with aerobic deterioration decreased in both dry stovers. We also observed that neither stover incurred any spoilage at 31°C after 120 days of exposure. The meteorological conditions affecting the survival of microbes clearly explain why the stover can be stored for such a long time during the dry season in West Africa.

In this study, the SS contained relatively low LBC, and abundant epiphytic LAB population. During ensiling, LAB can use sugars to increase the production of lactic acid, thereby reducing the pH and inhibiting the growth of harmful bacteria, which results in good‐quality SS silage. On the other hand, the PMS silage had a relatively low LAB in the stover could not ferment sufficient sugar to produce lactic acid. Furthermore, the pH of all the silages of both stovers did not decline below 4.20 and did not allow butyric acid fermentation and much NH_3_‐N production by clostridia. The low moisture in both silages may effectively inhibit the growth of clostridia, and SS is rich in LAB, which can promote lactic acid fermentation. Therefore, the fresh stovers can be prepared as silage, which is a good strategy for remedying the feed shortage for dairy cows in semi‐arid West Africa.

## 5 CONCLUSIONS

The chemical composition of PMS and SS during field exposure and silage fermentation were studied in Burkina Faso. The fresh PMS and SS contained various amounts of nutrients, silage fermentation preserved these nutrients well for a long term. With increased exposure time, the CP, EE, and GE contents of dry stovers decreased while the ADF and ADL contents increased sustainably. Therefore, silage technique using local available crop by‐product resources is an ideal strategy for relieving feed shortage in semi‐arid West Africa.

## CONFLICT OF INTEREST

We certify that there is no conflict of interest with any financial organization regarding the material discussed in the manuscript.
